# Choline Supplementation Modifies the Effects of Developmental Alcohol Exposure on Immune Responses in Adult Rats

**DOI:** 10.3390/nu14142868

**Published:** 2022-07-13

**Authors:** Jessica A. Baker, Kristen R. Breit, Tamara S. Bodnar, Joanne Weinberg, Jennifer D. Thomas

**Affiliations:** 1 Center for Behavioral Teratology, San Diego State University, San Diego, CA 92120, USA; jbaker3@sdsu.edu (J.A.B.); kbreit@wcupa.edu (K.R.B.); 2 Department of Psychology, West Chester University, West Chester, PA 19383, USA; 3 Department of Cellular and Physiological Sciences, Faculty of Medicine, University of British Columbia, Vancouver, BC V6T1Z3, Canada; tamara.bodnar@ubc.ca (T.S.B.); joanne.weinberg@ubc.ca (J.W.)

**Keywords:** ethanol, choline, cytokine, neuroimmune, immune, lipopolysaccharide, hippocampus, plasma

## Abstract

Prenatal alcohol exposure can disrupt the development of numerous systems, including the immune system. Indeed, alterations in cytokine levels may contribute to the neuropathological, behavioral, and cognitive problems, and other adverse outcomes observed in individuals with fetal alcohol spectrum disorders. Importantly, supplementation with the essential nutrient choline can improve performance in hippocampal-dependent behaviors; thus, the present study examined the effects of choline on plasma and hippocampal cytokines in adult rats exposed to ethanol in early development. From postnatal day (PD) 4–9 (third trimester equivalent), pups received ethanol (5.25 g/kg/day) or Sham intubations. Subjects were treated with choline chloride (100 mg/kg/day) or saline from PD10–30. On PD60, plasma and hippocampal tissue was collected before and after an immune challenge (lipopolysaccharide (LPS); 50 ug/kg). Prior to the immune challenge, ethanol-exposed subjects showed an overall increase in hippocampal pro-inflammatory cytokines, an effect mitigated by choline supplementation. In contrast, in the plasma, choline reduced LPS-related increases in pro-inflammatory markers, particularly in ethanol-exposed subjects. Thus, early choline supplementation may modify both brain and peripheral inflammation. These results suggest that early choline can mitigate some long-term effects of ethanol exposure on hippocampal inflammation, which may contribute to improved hippocampal function, and could also influence peripheral immune responses that may impact overall health.

## 1. Introduction

Fetal alcohol spectrum disorders (FASD) pose a serious public health concern across the globe, including in the United States, where they affect approximately 2–7% of children [1,2,3]. Alcohol exposure during gestation can disrupt normal development, resulting in a wide range of physical, neuropathological, cognitive, and behavioral alterations that present in childhood and can persist throughout a person’s life [4]. Emerging evidence indicates alterations in immune and neuroimmune systems after developmental alcohol exposure, including microglia activation (the brain’s resident immune cells) and cytokine dysregulations, which may contribute to impaired health and FASD neuropathology, as well as cognitive, behavioral, and other adverse outcomes [5]. Proper function of the immune/neuroimmune systems is crucial for normal brain development, as cytokines are involved in multiple developmental processes such as neurogenesis, myelination, angiogenesis, synaptogenesis, and apoptosis [6]. Interestingly, abnormalities in immune function have been linked to other neurodevelopmental disorders such as schizophrenia [7] and autism spectrum disorders [8], as well as neurodegenerative diseases, including Alzheimer’s disease [9].

Animal models of FASD have shown ethanol-induced neuroinflammation in multiple brain regions, including the hippocampus. Multiple studies have reported cytokine dysregulation leading to an imbalance of pro- and anti-inflammatory cytokines, as well as microglia activation in neonates exposed to alcohol during development [10,11,12,13,14]. Importantly, changes in neuroimmune activity after prenatal exposure to alcohol are not only seen in neonates, but may be long lasting [15,16]. Many studies report altered immune responses in subjects that were exposed to alcohol prenatally or during early postnatal life. For example, adult offspring with developmental alcohol exposure show exaggerated neuroimmune responses to the challenge of lipopolysaccharide (LPS) [14,15] or chronic adult ethanol exposure [17]. Interestingly, subjects that were prenatally exposed to alcohol and challenged with LPS as adults showed significant memory impairments, as well as increased hippocampal and peripheral pro-inflammatory cytokines, compared to controls [14].

Clinical studies have also shown that alcohol use during pregnancy can alter cytokine levels. Similar to animal studies, an initial clinical study reported increased levels of pro-inflammatory cytokines, including TNF-α, IL-1β, and IL-6, in maternal and fetal blood collected from chronic alcohol users during pregnancy [18]. More recent studies have identified specific cytokine networks in both maternal and child blood samples that are associated with both alcohol consumption during pregnancy and adverse child neurodevelopment [19,20]. Collectively, these preclinical and clinical studies suggest exposure to alcohol during development elicits long-lasting changes in immune and neuroimmune function. Such alterations may contribute to the developmental changes seen in FASD.

Identification of treatments that reduce the severity of FASD is critical, specifically regarding interventions that can improve neurodevelopment and cognitive function. One potential intervention that is currently being investigated in FASD is the essential nutrient choline [21]. Choline is critical for normal brain development and cognitive function, with studies showing life-long memory enhancement in typically developing subjects that received perinatal choline supplementation [22,23,24,25,26,27,28]. Preclinical studies have illustrated that perinatal choline supplementation is also neuroprotective in a variety of both neurodevelopmental and neurodegenerative models [27,28]. Although some choline is produced endogenously, choline levels depend largely on dietary intake [24,25].

Preclinical models have demonstrated that choline supplementation can attenuate the effects of developmental alcohol exposure, specifically alcohol-induced behavioral and cognitive alterations [29,30,31,32,33,34]. Moreover, choline can improve hippocampal-dependent learning and memory [29,30,31,34] and can enhance hippocampal plasticity [35], even when administered after developmental ethanol exposure. Recent clinical studies in FASD support preclinical findings, with cognitive improvements reported in children who received choline supplementation either prenatally [36,37] or postnatally [38,39]. Notably, one recent clinical trial found toddlers with FASD that received choline supplementation for 9 months showed improvements in visual–spatial skills, working memory, and attention 4 years after choline supplementation was provided [38,39]. These studies demonstrate the ability of choline to attenuate alcohol-induced behavioral and cognitive alterations, inducing long-lasting beneficial effects.

Although the mechanisms behind the beneficial effects of choline in FASD are not well understood, it is possible that choline may reduce the severity of FASD, in part, by mitigating alcohol-induced immune/neuroimmune dysregulation. Choline acts as an anti-inflammatory modulator in neurodegenerative [40,41] and neurodevelopmental [42] models. Choline is a selective competitive agonist for alpha-7 nicotinic acetylcholine receptors (α7AChR) that are found on both neurons and microglia in various brain regions, including the hippocampus. Importantly, preclinical models show that choline can affect cytokine levels during development. For example, a human placental trophoblast cell-culture model found that choline deficiency significantly increased expression of multiple pro-inflammatory cytokines [43]. In contrast, prenatal choline supplementation can reduce the expression of pro-inflammatory cytokines, such as TNF-α and IL1-β, in both typical [44] and atypical developmental models [45].

Choline supplementation may also modulate immune and neuroimmune responses to environmental stimuli. Prenatal choline supplementation can reduce immune responses, as measured by inflammatory cytokines in serum and placenta, in pregnant rats exposed to LPS during gestation [46]. Not only can choline modify peripheral immune responses, but choline supplementation can also reduce LPS-induced pro-inflammatory cytokines in the hippocampus and protect against LPS-induced cognitive impairments [47]. Moreover, in a maternal immune activation mouse model (a model of environmental risk factor for autism and schizophrenia), maternal choline supplementation triggered an anti-inflammatory response in fetal brains and improved behavioral alterations when these subjects became adults [42]. Collectively, these studies suggest that choline could mitigate alcohol-induced immune and neuroimmune dysregulation.

The current study examined the effects of postnatal choline supplementation on peripheral and hippocampal cytokines in a rodent model that simulates the third trimester of human pregnancy. The purpose of this study was to (1) examine the effects of choline supplementation on baseline cytokine levels in adult animals exposed to alcohol during early development, and (2) determine the ability of choline supplementation to modulate LPS-induced immune and neuroimmune responses in alcohol-exposed subjects.

## 2. Materials and Methods

### 2.1. Subjects

All experimental procedures were approved by the San Diego State University (SDSU) Institutional Animal Care and Use Committee in accordance with the National Institutes of Health’s *Guide for the Care and Use of Laboratory Animals.* Adult male and female Sprague Dawley rats were obtained from Charles River Laboratories (Hollister, CA, USA). Subjects used in this study were bred in the mating colony at the SDSU Center for Behavioral Teratology vivarium. One male and one female Sprague Dawley rat were housed overnight, and the presence of a seminal plug indicated gestational day (GD) 0. Pregnant dams were then singly housed in a temperature- and humidity-controlled environment with the day of birth, usually occurring on GD 22, being designated as the postnatal day (PD) 0. On PD 1, litters were culled to 8 pups (4 males and 4 females when possible). On PD 7, pups had their paw pads tattooed with non-toxic veterinary black tattoo ink (STONE Manufacturing and Supply Company, Kansas City, MO, USA) for identification purposes that kept the experimenters blind to treatment. Pups were weaned on PD 21 and separated by sex on PD 28.

### 2.2. Design and Treatment

**Figure 1:**
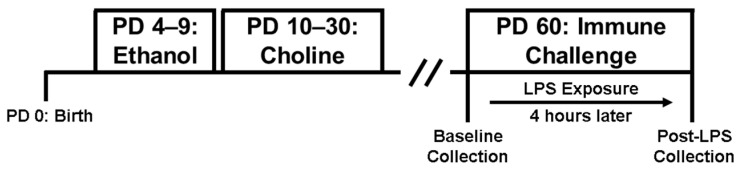
Overview of experimental design. Timeline and overview exposure groups: ethanol exposure from postnatal day (PD) 4–9 (**left**), choline supplementation from PD 10–30 (**middle**), and immune challenge of lipopolysaccharide (LPS) on PD 60 (**right**).

The current study used a 2 Ethanol (Ethanol (EtOH), Sham) × 2 Choline (Choline, Saline) × 2 Sex (Male, Female) design. On PD 4, neonatal rat pups were randomly assigned to an exposure (Ethanol, Sham) and treatment (Choline, Saline) group. One sex pair per litter was assigned to each group to control for potential litter effects. Figure 1 shows the experimental timeline.

### 2.3. Developmental Alcohol Exposure

On PD 4–9, rats were developmentally exposed to ethanol during their brain growth spurt; this period mimics 3rd-trimester development in humans [48], when the hippocampus is particularly vulnerable to alcohol exposure [49,50]. Subjects assigned to the ethanol exposure group received 5.25 g/kg/day of ethanol (Sigma Aldrich, St. Louis, MO, USA), dissolved in an artificial milk diet [51]. The ethanol–milk solution (11.9% *v*/*v*) was delivered in 2 intragastric intubations 2 h apart. Two additional milk feedings (2 h apart) were given each day [52]. This method of binge-like developmental alcohol exposure produces blood alcohol concentrations (BAC) consistent with high alcohol consumption [52], comparable to moderate–high binge drinking levels in humans [53]. Subjects assigned to the Sham control group did not receive ethanol and/or milk diet. However, they did undergo the same number of intragastric intubations (Sham intubations).

### 2.4. Peak Blood Alcohol Concentrations

On PD 6, 90 min after the second ethanol intubation, 20 µL of blood was collected from the tail to determine peak BAC [54]. Plasma was analyzed using an Analox Alcohol Analyzer (Model AM1, Analox Instruments, Lunenburg, ME, USA). Sham controls also had blood collected to help control for potential procedural stress.

### 2.5. Choline Supplementation

From PD 10–30, half of the subjects in each of the exposure groups were injected subcutaneously (s.c.) with 100 mg/kg/day of choline (6.667 mL/kg/day; Balchem, New Hampton, NY, USA). This dose is the most effective at reducing behavioral alterations associated with 3rd-trimester alcohol exposure [30]. The remaining subjects received injections of sterile saline (0.85% sodium chloride solution, Sigma-Aldrich, St. Louis, MO, USA). This time period in rats is the equivalent of early infancy and childhood in humans [55].

### 2.6. Immune Challenge and Tissue Collection

On PD 60, rats received an intraperitoneal (i.p.) injection of 50 µg/kg of lipopolysaccharide (LPS; Escherichia coli; Serotype 026:B6, Sigma Aldrich, St. Louis, MO, USA), dissolved in sterilized saline (2 mL/kg), as an immune challenge. Blood samples were collected from the tail 30 min prior to the immune challenge (pre-LPS) and 90 min post-injection (post-LPS). Four hours following the immune challenge, rats were anesthetized using isoflurane and 600 µL of blood was collected via intracardial puncture prior to euthanasia via decapitation. Brains were removed and hippocampi were dissected and flash frozen using liquid nitrogen. Samples were transported on dry ice to the University of British Columbia for further processing.

### 2.7. Tissue Homogenization

Lysis buffer was prepared containing 150 mM NaCl, 20 mM Tris pH 7.5, 1 mM EDTA, 1 mM EGTA, and 1% Triton X-100, and immediately prior to homogenization, the following were added (per 10 mL lysis buffer): 1 complete mini protease inhibitor cocktail tablet (Roche Diagnostics, Indianapolis, IN, USA), 100 µL phosphate inhibitor 2 and 3 (Sigma-Aldrich, St. Louis, MO, USA), 100 µL 1 M NaF, and 40 µL PMSF (from 500 mM stock in DMSO). Hippocampal samples were added to 1.6 mL tubes containing 8 zirconium oxide beads and 200 µL lysis buffer. Samples were homogenized using the Omni Bead Ruptor 24 (Omni International, Kennesaw, GA, USA) in 4 cycles (speed: 2.10, time: 5 s), with 1 min on ice in between cycles. Following homogenization, tissue samples were centrifuged at 1400× *g* for 10 min at 4 °C. Separate aliquots of supernatant were removed for protein quantification and cytokine analyses and stored at −20 °C until assayed.

### 2.8. Protein Measurements

Total protein levels were quantified in tissue hippocampal homogenates using the Pierce Microplate BCA Protein Assay Kit (Pierce Biotechnology, Rockford, IL, USA). Tissue homogenates were diluted (1:41) and the standard BCA protocol was followed. Homogenate samples were run in quadruplicate and the average protein concentration (µg/mL) across the 4 samples was calculated (samples with CVs ≥ 10 were re-run). Cytokine levels were then adjusted, and values reported as pg cytokine/mg of protein.

### 2.9. Cytokine Measurements

Multiplex cytokine assays were performed using the Meso Scale Discovery (MSD) pro-inflammatory panel 1 rat V-PLEX kit (Meso Scale Diagnostics, Rockville, MD, USA). This 9-plex cytokine panel allows for the simultaneous measurement of IL-1β, IL-4, IL-5, IL-6, IL-10, IL-13, IFN-ɣ, KC/GRO (CXCL1), and TNF-α (catalog #: K15059D, Meso Scale Diagnostics, Rockville, MD, USA). Samples were diluted in diluent 42 (1:2 dilution) and assays were performed using the standard MSD protocol. Cytokine plates were read using a Sector Imager 2400 (Meso Scale Diagnostics, Rockville, MD, USA) and data analyzed using the MSD Discovery Workbench software v. 4.0 (Meso Scale Diagnostics, Rockville, MD). The lower limit of detection (LLOD) for the plasma samples was: (pg/mL): IL-1β: 8.06; IL-4: 0.40; IL-5: 9.95; IL-6: 22.40; IL-10: 4.20; IL-13: 0.62; IFN-ɣ: 0.58; TNF-α: 0.40; KC/GRO: 0.56; and hippocampal samples (pg/mg): IL-1β: 9.58; IL-4: 0.78; IL-5: 12.30; IL-6: 52.90; IL-10: 2.37; IL-13: 0.99; IFN-ɣ: 1.26; TNF-α: 0.48; KC/GRO: 0.86. Cytokines levels falling below the LLOD were assigned a value of 0.

### 2.10. Statistical Analyses

Dependent variables were plasma cytokine levels (pg/mL), percent change in plasma cytokine levels (change from pre-LPS to post-LPS), hippocampal cytokine levels (pg/mg), and hippocampal cytokine difference scores (baseline hippocampal cytokine average per group subtracted from LPS hippocampal cytokine level). Heatmaps and dendrograms were used to visualize overall cytokine profiles across groups (averaged per group; built on z-scored data), and were created using R software (version 4.2, R Foundation for Statistical Computing, Vienna, Austria) and the gplots package (version 3.1.3).

Outliers were identified using a z-score analysis (>|3.29|) and were Winsorized prior to statistical analysis. Cytokine levels were Blom transformed for statistical analysis [56]. IL-4 levels were undetectable in a majority of the samples, and were excluded from further analysis, with the exception of the heatmaps and dendrograms. Data that were normally distributed were analyzed using a 2 (Ethanol, Sham) × 2 (Choline, Saline) × 2 (Male, Female) Analysis of Variance (ANOVA) using the Statistical Packages for Social Sciences (SPSS; version 28.0.1.0, IBM, Armonk, NY, USA). For plasma data, repeated measures ANOVAs were used when applicable. Post hoc analyses were conducted using Fisher’s Least Significant Difference (LSD) Tests. Data that did not pass normality tests were analyzed using non-parametric analyses; these examined the main effects of Ethanol, Choline, and Sex (Mann–Whitney U) or group effects (Kruskal–Wallis H: Ethanol + Choline, Ethanol + Saline, Sham + Choline, Sham + Saline), with Chi-Square analyses for individual follow-up comparisons. Means (M) and standard errors of the mean (SEM) are reported when applicable. All significance levels were set as *p* < 0.05 and trends towards significance were set as *p* < 0.10. All graphs illustrate the Winsorized data.

## 3. Results

### 3.1. Blood Alcohol Concentrations

Mean ± SEM blood alcohol concentrations were 296 ± 8 mg/dL for ethanol-exposed subjects treated with choline and 304 ± 8 mg/dL for ethanol-exposed saline controls. There was no significant difference in blood alcohol concentrations between the two ethanol-exposed groups (*F*_(1,37)_ = 0.4, *p* = 0.51).

### 3.2. Hippocampus Cytokine Levels at Baseline and after LPS Challenge

#### 3.2.1. Hippocampal Cytokine Profile

**Figure 2:**
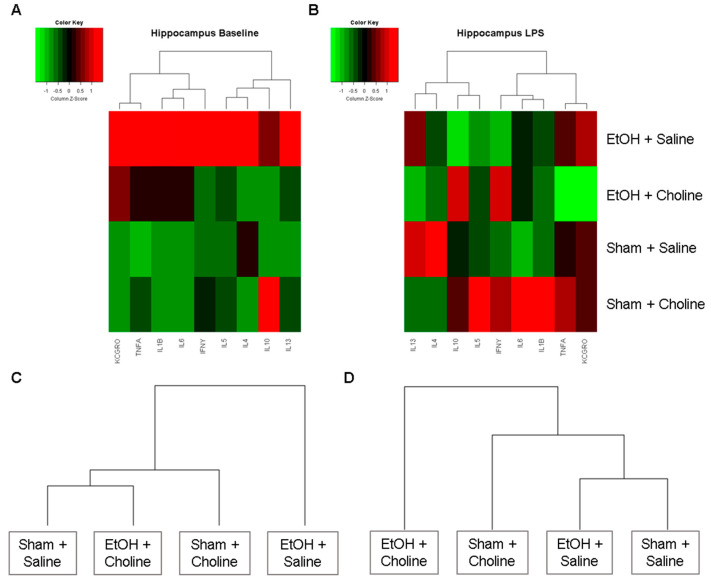
Overall hippocampal cytokine profiles. Heatmaps depicting overall cytokine profiles in the hippocampus in each group at (**A**) baseline and (**B**) after LPS immune challenge. Rows represent treatment groups and columns represent hierarchical clustering of mean cytokine levels (z-scored data). Additional dendrograms depict clustering of the treatment groups based on hippocampal cytokine profiles at (**C**) baseline and (**D**) after LPS immune challenge.

Heatmaps were produced to examine overall cytokine patterns in the hippocampus across the four treatment groups: (1) Ethanol + Saline, (2) Ethanol + Choline, (3) Sham + Saline, (4) Sham + Choline (Figure 2). At baseline, overall hippocampal cytokine profiles were strikingly different between ethanol-exposed animals that did not receive choline supplementation and all other groups (Figure 2A), with the highest mean cytokine levels in the ethanol-exposed animals. After LPS exposure, groups that received choline exhibited different overall hippocampal cytokine profiles were compared to comparable groups that did not receive choline (Figure 2B). Interestingly, when clustering groups based on their overall hippocampal cytokine profiles, ethanol-exposed subjects that received choline were more similar to control animals (with or without choline supplementation) at baseline (Figure 2C). However, after the immune challenge, subjects that did not receive choline were more similar to one another than to choline-treated groups (Figure 2D).

#### 3.2.2. Hippocampal Cytokine Levels

**Figure 3:**
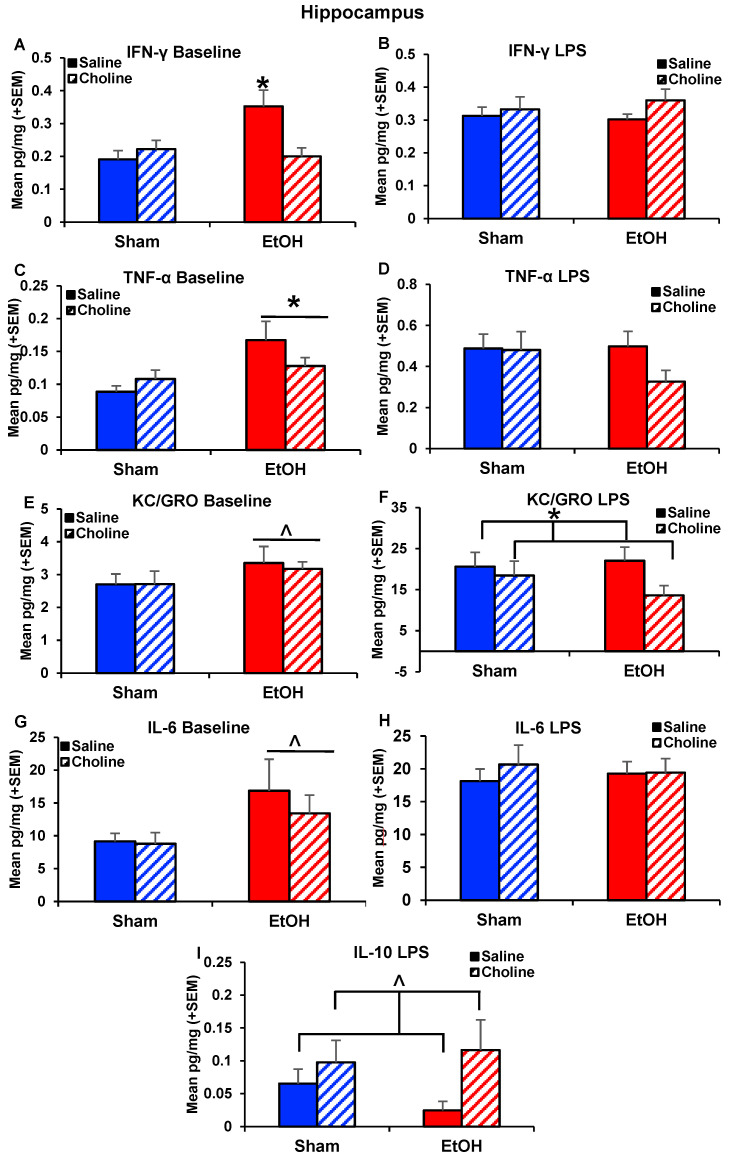
Hippocampal cytokine levels at baseline and following immune challenge of LPS. The following hippocampal cytokines are depicted in the graphs above: (**A**) Baseline IFN-ɣ (EtOH + Choline < EtOH + Saline, * *p* < 0.05), (**B**) LPS IFN-ɣ, (**C**) Baseline TNF-α (EtOH > Sham, * *p* < 0.05), (**D**) LPS TNF-α, (**E**) Baseline KC/GRO (EtOH > Saline, ^ *p* < 0.10), (**F**) LPS KC/GRO (Choline < Saline, * *p* = 0.05), (**G**) Baseline IL-6 (EtOH > Saline, ^ *p* < 0.10), (**H**) LPS IL-6, and (**I**) LPS IL-10 (Choline > Saline, ^ *p* < 0.10). n = 19–24 per group.

These overall patterns are further illustrated with examination of individual cytokine levels. At baseline, subjects exposed to ethanol had significantly heightened levels of IFN-ɣ (*F*_(1,41)_ = 7.49, *p* < 0.01; Figure 3A) and TNF-α (*F*_(1,79)_ = 6.85, *p* < 0.05; Figure 3C), with similar trends for increases in KC/GRO (*F*_(1,79)_ = 3.07, *p* < 0.08; Figure 3E) and IL-6 (*U* = 750, *p* < 0.10; Figure 3G). Importantly, choline supplementation mitigated ethanol-related elevations in IFN-ɣ, producing a significant interaction of Ethanol*Choline (*F*_(1,79)_ = 6.13, *p* < 0.05; Figure 3A). Following the adult LPS challenge, the hippocampal cytokine response did not differ in ethanol-exposed subjects. However, choline supplementation significantly reduced the pro-inflammatory chemokine KC/GRO (*F*_(1,77)_ = 3.85, *p* < 0.05; Figure 3F) and tended to increase the anti-inflammatory cytokine IL-10 (*U* = 737.00, *p* = 0.08,; Figure 3I). There were no sex effects at baseline; there were a few main effects of sex following LPS challenge, but significant interactions of sex with other variables.

**Figure 4:**
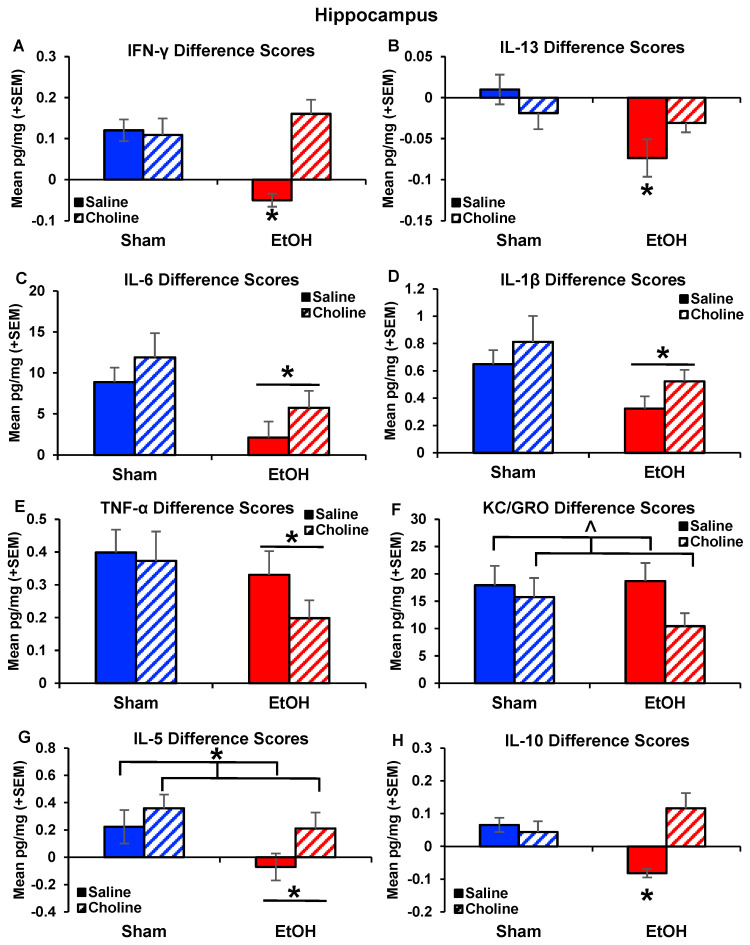
LPS-induced changes in hippocampal cytokines. Difference scores of cytokines after an immune challenge of LPS. There was an effect of ethanol exposure or choline supplementation on the following change in levels after LPS exposure for the following cytokines: (**A**) IFN-ɣ (EtOH + Choline > EtOH + Saline, * *p* < 0.05), (**B**) IL-13 (EtOH + Choline > EtOH + Saline, * *p* < 0.05), (**C**) IL-6 (EtOH < Sham, * *p* < 0.05), (**D**) IL-1β (EtOH < Sham, * *p* < 0.05), (**E**) TNF-α (EtOH < Sham, * *p* < 0.05), (**F**) KC/GRO (Choline < Saline, ^ *p* < 0.10), (**G**) IL-5 (Choline > Saline, * *p* < 0.05 and EtOH < Sham * *p* < 0.05), and (**H**) IL-10 (EtOH + Choline > EtOH + Saline, * *p* < 0.05). n = 21–22 per group.

Difference scores were calculated to further examine cytokine responses after immune challenge while accounting for group differences at baseline. Interestingly, there were significant interactions between ethanol exposure and choline on IFN-ɣ (*F*_(1,77)_ = 21.07, *p* < 0.001; Figure 4A), as well as the main effects of ethanol (*F*_(1,77)_ = 6.46, *p* < 0.05), and choline (*F*_(1,77)_ = 12.21, *p* < 0.001). Subjects exposed to ethanol without choline supplementation differed significantly compared to all other groups (*p*’s < 0.05). In fact, there was no increase in IFN-ɣ following LPS challenge in the ethanol-exposed group, whereas ethanol-exposed subjects that received choline supplementation showed significant increases IFN-ɣ level after LPS exposure, similar to non-exposed controls (*p* < 0.05).

Similar effects were observed in the anti-inflammatory cytokine IL-13, with an interaction of Ethanol*Choline (*F*_(1,77)_ = 19.18, *p* < 0.001; Figure 4B), as well as the main effects of ethanol (*F*_(1,77)_ = 19.30, *p* < 0.05), choline (*F*_(1,77)_ = 7.15, *p* < 0.01), and sex (*F*_(1,77)_ = 7.15, *p* < 0.01). In addition, there were significant interactions of Ethanol*Choline*Sex (*F*_(1,77)_ = 4.87, *p* < 0.05) and Ethanol*Sex (*F*_(1,77)_ = 17.24, *p* < 0.001). Choline supplementation mitigated ethanol-induced reductions in IL-13 after LPS in both male and female subjects (*p* < 0.05), producing significant interactions between ethanol and choline in both sexes. The pattern was more robust among females compared to males (Supplemental Figure S1).

Ethanol exposure also significantly blunted IL-6 (*F*_(1,77)_ = 8.84, *p* < 0.01, Figure 4C) and IL-1β (*F*_(1,77)_ = 4.69, *p* < 0.05; Figure 4D) responses after LPS exposure compared to non-ethanol-exposed subjects. Although the effects of choline failed to reach statistical significance, it is notable that levels of IL-1β and IL-6 in ethanol-exposed subjects treated with choline did not differ from that of either control group. Ethanol-exposed subjects also exhibited reduced TNF-α responses after LPS exposure (*F*_(1,77)_ = 5.47, *p* < 0.05; Figure 4E) compared to non-exposed controls. Although ethanol exposure did not affect KC/GRO responses, choline supplementation (*F*_(1,77)_ = 3.00, *p* = 0.08; Figure 4F) tended to reduce the response after LPS exposure.

The difference scores for the anti-inflammatory cytokines, IL-5 and IL-10, showed similar effects of ethanol exposure and choline supplementation. There was less of an IL-5 response to LPS in ethanol-exposed subjects compared to non-exposed subjects (*F*_(1,77)_ = 6.90, *p* < 0.01; Figure 4G), an effect driven by the ethanol-exposed subjects that were not treated with choline. In addition, responses were greater among choline-treated subjects compared to non-supplemented subjects (*F*_(1,77)_ = 5.67, *p* < 0.05; Figure 4G). Although the interaction of ethanol and choline did not reach statistical significance, ethanol-exposed subjects that received choline supplementation had greater increases in IL-5 after LPS exposure compared to ethanol-exposed subjects that did not receive choline (*p* < 0.01). Similar effects were seen for IL-10 (Figure 4H). Few subjects had detectable levels of IL-10 at baseline (94% undetectable); however, all groups showed an increase in the number of subjects with detectable levels post-LPS (Fisher exact probabilities *p* < 0.05), except the ethanol-exposed group without choline. Non-parametric analyses confirmed differences that ethanol-exposed subjects without choline had less of an IL-10 response compared to all other groups, including controls (*X*^2^_(13,42)_ = 42, *p* < 0.001) and ethanol-exposed subjects that received choline supplementation (*X*^2^_(13,42)_ = 42, *p* < 0.001).

### 3.3. Plasma Cytokine Levels Pre- and Post-LPS Challenge

#### 3.3.1. Plasma Cytokine Profile

**Figure 5:**
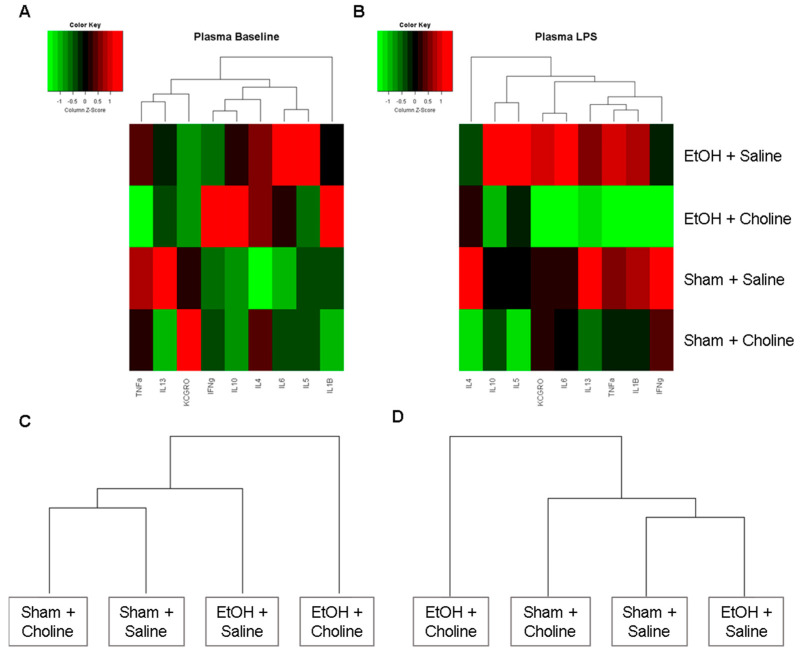
Overall plasma cytokine profiles. Heatmaps depicting overall cytokine profiles in the plasma in each group at (**A**) baseline and (**B**) after LPS immune challenge. Rows represent treatment groups and columns represent hierarchical clustering of mean cytokine levels (z-scored data). Additional dendrograms depict clustering of the treatment groups based on plasma cytokine profiles at (**C**) baseline and (**D**) after LPS immune challenge.

In contrast with the hippocampal cytokine profiles, heatmaps of plasma cytokines showed that treatment differences were more evident following the immune challenge rather than at baseline (Figure 5). At baseline, overall plasma cytokine profiles show more similarities between ethanol-exposed groups compared with non-exposed groups, regardless of choline supplementation (Figure 5A,C). However, after an LPS challenge, there was a visible difference in plasma cytokine profiles with ethanol-exposed subjects that received choline showing overall reductions in cytokines compared to ethanol-exposed subjects that were not treated with choline (Figure 5B). Hierarchal clustering showed that groups that did not receive choline supplementation (with or without ethanol exposure) were more similar to one another than those that received choline supplementation (Figure 5D).

#### 3.3.2. Plasma Cytokine Levels

**Figure 6:**
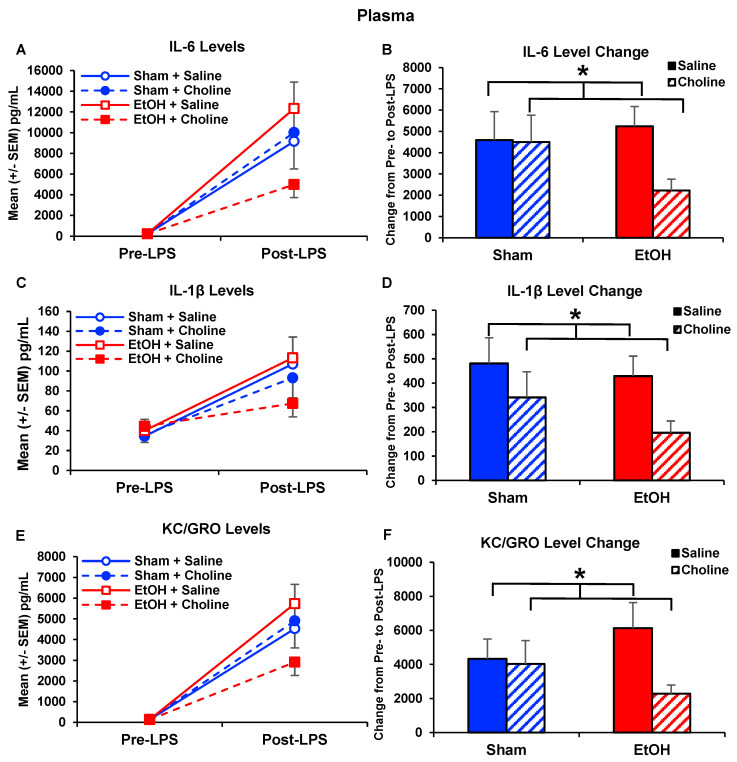
Early postnatal choline treatment reduces plasma cytokine release following immune challenge of LPS. There was a significant effect of choline supplementation, which reduced LPS-induced response in (**A**,**B**) IL-6 (Choline < Saline, * *p* < 0.05), (**C**,**D**) IL-1β (Choline < Saline, * *p* < 0.05), and (**E**,**F**) KC/GRO (Choline < Saline, * *p* < 0.05), an effect that was largely driven by ethanol-exposed subjects. n = 21–22 per group.

Data illustrate that early postnatal choline treatment reduces select cytokine levels following an immune challenge. Choline reduced IL-6 (*F*_(1,77)_ = 4.19, *p* < 0.05; Figure 6A,B), IL-1β (*U* = 611.50, *p* < 0.05; Figure 6C,D), and KC/GRO (*F*_(1,77)_ = 4.21, *p* < 0.05; Figure 6E,F) elevations caused by LPS challenge. Although the interactions of ethanol × choline did not reach statistical significance, these effects were driven by choline-treated subjects exposed to alcohol.

**Figure 7:**
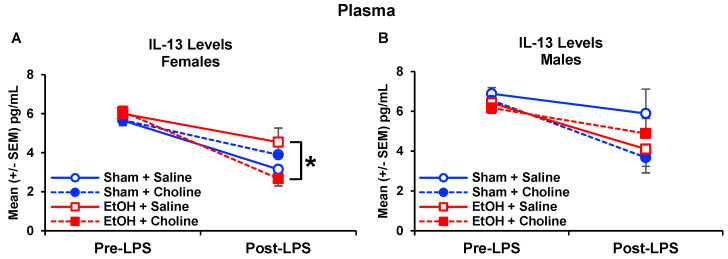
Sex differences in IL-13 plasma cytokine levels. (**A**) Females exposed to ethanol and treated with choline had lower IL-13 post-LPS (EtOH + Choline < EtOH + Saline, * *p* < 0.05). (**B**) No effects of ethanol exposure or choline supplementation were observed among males. n = 10–11 per group.

**Figure 8:**
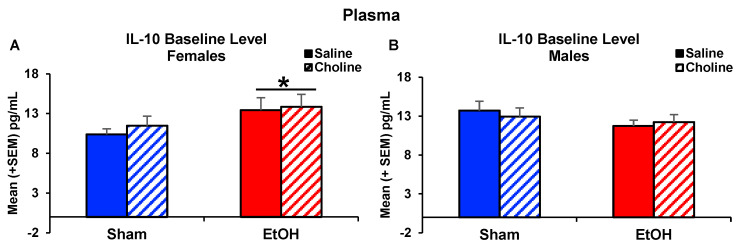
Sex differences in IL-10 plasma cytokines levels. Females exposed to ethanol during early development had lower baseline levels of IL-10. (**A**) Baseline IL-10 levels in females (EtOH > Sham, * *p* < 0.05). (**B**) Baseline IL-10 levels in males. n = 10–11 per group.

Among plasma cytokine levels, there were some main effects of sex, with interactions with sex with other variables present in only two cytokines, IL-13 and IL-10. Interactive effects of developmental ethanol exposure, choline supplementation, and sex were observed in plasma IL-13 levels, yielding a four-way interaction of Time*Sex*Ethanol*Choline (*F*_(1,77)_ = 4.37, *p* < 0.05; Figure 7). At baseline, males had higher IL-13 levels compared to females (*F*_(1,77)_ = 11.33, *p* < 0.01), regardless of ethanol exposure or choline supplementation. Post-LPS administration, ethanol-exposed females who were supplemented with choline postnatally had lower levels of IL-13 levels compared to ethanol-exposed females only treated with saline (*p* < 0.05; Figure 7A), producing a three-way interaction of Sex*Ethanol*Choline (*F*_(1,77)_ = 6.88, *p* < 0.05). Among IL-10 levels, a three-way interaction of Time*Sex*Ethanol was observed (*F*_(1,77)_ = 4.06, *p* < 0.05, Figure 8). At baseline, female subjects exposed to ethanol during early development had greater IL-10 levels than their Sham-intubated counterparts (*F*_(1,39)_ = 4.45, *p* < 0.05; Figure 8A), yielding a Sex*Ethanol interaction (*F*_(1,77)_ = 5.72, *p* < 0.05), whereas no differences were observed among male subjects (Figure 8B). However, this effect was not observed post-LPS administration, and the percent change data also yielded no significant differences.

## 4. Discussion

The current study aimed to examine the effects of choline supplementation on peripheral and hippocampal cytokine levels in adult animals that were exposed to alcohol developmentally; animals were assessed both at baseline and after immune challenge. We found unique effects of ethanol exposure and choline supplementation on cytokine levels in the hippocampus and plasma. When examining the overall hippocampal cytokine profile, ethanol-exposed subjects generally exhibited elevated mean cytokine levels at baseline, such as IFN-ɣ, IL-6, TNF-α, and KC/GRO, with choline supplementation mitigating many ethanol-induced cytokine changes. In contrast, the overall plasma cytokine profile showed that choline generally blunted cytokine responses to an immune challenge, including IL-1β, IL-6, and KC/GRO. These results demonstrate that developmental alcohol exposure and early choline treatment have differential effects on the immune and neuroimmune system.

There is a large body of literature examining neuroimmune responses following adult or adolescent alcohol exposure [57,58,59]; however, the effects of developmental alcohol exposure on the neuroimmune system are understudied. In fact, only a handful of studies have examined the long-term effects (in adults) of developmental alcohol exposure on cytokine levels in the brain. In the present study, we identified several cytokines in the hippocampus that showed elevated levels at baseline (pre-immune challenge) in adult subjects exposed to ethanol early in development, including pro-inflammatory cytokines TNF-α, KC/GRO, and IFN-ɣ. Many of these cytokines, including TNF-α, IL-6, and IFN-ɣ, have previously been found to be elevated in the hippocampus in neonatal subjects exposed to either prenatal or postnatal (third trimester equivalent of human pregnancy) ethanol [10,11,12,13,60,61]. Our results indicate that such effects are long lasting, consistent with a previous study that found increased hippocampal IL-6 expression in adults prenatally exposed to ethanol [62]. Others have reported long-lasting effects of prenatal ethanol exposure on TNF-α, although they found reductions, rather than elevations, in ethanol-exposed subjects [17]. Collectively, these data indicate that there is a change in the hippocampal inflammatory state that is evident long after ethanol exposure.

Importantly, we found that ethanol-induced increases in IFN-ɣ were significantly mitigated by choline supplementation. In both physiological and pathological conditions, cytokine can be secreted from numerous cell types in the hippocampus, including neurons, microglia, astrocytes, and infiltrating peripheral immune cells (as reviewed in [63]). Increased microglial activation has been reported shortly after early alcohol exposure [11,12,13], but we have not yet elucidated the source of long-lasting changes in the cytokine within our model. IFN-ɣ levels in the hippocampus have been linked to hippocampal function and cognitive performance, with IFN-ɣ knockout models reporting increased neurogenesis, hippocampal plasticity, and improved learning and memory [64]. Moreover, increases in hippocampal IFN-ɣ can impair cognitive function, reduce neurogenesis, and increase other cytokines, including TNF-α and IL-6 [65]. Importantly, in the current study, ethanol-exposed subjects that were given choline supplementation showed reduced IFN-ɣ levels that were similar to those of controls. Not only was choline supplementation able to rescue ethanol-induced increases in IFN-ɣ, even when given *after* the ethanol exposure; these changes were also long lasting, evident in adults long after choline supplementation had ceased. This mitigation of the ethanol effects is consistent with studies showing that postnatal choline supplementation improves hippocampal-dependent learning and memory following developmental alcohol exposure, using the same alcohol and choline treatment parameters as in the present study [29,30,31,32,33,34].

Developmental alcohol exposure has also been shown to affect neuroimmune markers in response to an immune challenge in adulthood (reviewed in [5,66]). Therefore, we examined the effects of developmental ethanol exposure and early choline supplementation on adult hippocampal cytokine levels after LPS exposure. We found that ethanol exposure blunted the LPS-induced cytokines release, specifically blunting the pro-inflammatory cytokines, IFN-ɣ, IL-6, TNF-α, and IL-1β, as well as the anti-inflammatory cytokines, IL-5, IL-6, IL-10, and IL-13. Importantly, choline supplementation was able to rescue most of the dampened cytokine responses to LPS challenge in ethanol-exposed subjects; specifically, ethanol-exposed subjects that received choline showed significant differences in LPS-induced levels of IFN-ɣ, IL-5, IL-10, and IL-13 compared to non-supplemented ethanol-exposed subjects. Levels of these cytokines in choline-supplemented ethanol-exposed subjects were similar to levels of controls. Likewise, although LPS-induced levels of IL-6 and IL-1β were significantly blunted in ethanol-exposed subjects, those that received choline did not differ from either control group. There have been limited studies on the effects of developmental alcohol on hippocampal cytokines in adults after an immune challenge. However, prenatal alcohol exposure has previously been reported to increase levels of TNF-α, IL-6, and IL-1β in response to an immune challenge of LPS [14] or alcohol exposure [17,67] in adult subjects. In contrast, Topper and Valenzuela (2014) found no effects of LPS on cytokines in the hippocampus of juveniles exposed to alcohol in the early postnatal period, but did find sex-dependent blunting of IL-1β expression in the frontal cortex [68]. Thus, although many studies suggest that prenatal alcohol exposure primes the neuroimmune system to be hyper-responsive to later immune challenges, the long-lasting effects may be variable. Regardless, these results show effects of developmental ethanol exposure on neuroimmune markers long after the ethanol insult.

In the plasma, there were limited effects of early ethanol exposure at baseline with increases in only one cytokine, IL-10, in females, regardless of choline supplementation. Although clinical studies have found alcohol exposure during pregnancy altered cytokine levels in maternal plasma [18,19,69], as well as the plasma of young children [20], to date, plasma cytokines have not been examined in adults exposed to alcohol prenatally. Similarly, limited preclinical studies have examined the effects of developmental ethanol exposure on plasma cytokines. A recent study reported no effects of prenatal alcohol exposure on cytokine levels in adult plasma, but did find altered cytokine profiles in other peripheral tissues (i.e., liver, spleen) [70]. Similarly, other preclinical studies have found limited effects of prenatal ethanol exposure on LPS-induced cytokine levels in adults [71,72].

At baseline, choline did not affect plasma cytokine levels. However, choline supplementation significantly blunted LPS-induced elevations of IL-6, IL-1β, and KC/GRO. Overall, choline-induced reductions in plasma cytokines after LPS exposure can be clearly seen in the heatmap of plasma LPS cytokine profiles (Figure 4B). These effects are similar to previous studies that also found choline supplementation reduced IL-1β and IL-6 plasma levels after LPS exposure [46,73,74]. Although there were no significant interactions between ethanol and choline treatments, examination of the data shows that these choline-related reductions in LPS-responses were driven by ethanol-exposed subjects.

In our current study, we show long-lasting effects of choline supplementation on both immune and neuroimmune systems. There are many possible mechanisms by which choline could act, including altered acetylcholine (ACh) neurotransmission, epigenetic modifications, and lipid metabolism [25,75]. Interestingly, there are known interactions between choline and immune cells, with choline acting as an anti-inflammatory modulator in many neurodegenerative and neurodevelopment models [40,41,42]. Choline is also a selective competitive agonist for α7nACh receptors that are found on both microglia and neurons. Activation of α7nACh receptors alters microglial cytokine production and release [76,77], as well as modulating memory and hippocampal plasticity [41]. Importantly, activation of α7nACh receptors can also affect peripheral immune cell function and modulate production of cytokines [78,79,80]. The ability of choline to activate α7nACh receptors is one possible mechanism behind the modulating effects of choline supplementation on immune and neuroimmune cytokine levels.

To date, few studies have examined the effects of developmental alcohol exposure on both peripheral and central cytokine levels [10,14,81]. As peripheral immune markers, such as plasma cytokine levels, are being investigated as potential biomarkers for fetal alcohol exposure [19,20], it is imperative to understand the long-term effects of prenatal alcohol exposure on peripheral cytokines. Moreover, it is important to examine the relationship between cytokine levels in the brain and the periphery, and to determine if plasma immune changes predict or reflect neuroimmune changes. In the present study, we found differential effects of developmental ethanol exposure in plasma and brain in adults both at baseline and after LPS challenge. Our results are similar to other studies that examined cytokines in both the brain and periphery in neonates exposed to alcohol prenatally, finding little to no overlap in cytokine levels between plasma and multiple other brain regions, including the hippocampus, prefrontal cortex, hypothalamus [10], and amygdala [81]. Given that clinical data are limited to peripheral markers, careful consideration must be given before inferring similar changes in the brain. Clearly, additional research is needed to elucidate the relationship between peripheral and central immune markers and better understand how both peripheral and central cytokines may influence neurodevelopmental alterations seen in FASD.

Currently, choline supplementation is being investigated as an intervention to help reduce the severity of FASD [21]. Many clinical studies have examined the beneficial effects of both prenatal and postnatal choline supplementation on neurobehavioral and cognitive function in children with FASD [36,37,38,39]. Importantly, these improvements are seen years after choline supplementation has ceased, indicating long-lasting effects in these children [39]. The present data suggest that alcohol exposure leads to long-lasting changes in hippocampal cytokine levels, and that choline may attenuate the altered inflammatory state of the hippocampus, potentially improving hippocampal function and cognitive outcomes. Considering that choline also modifies plasma cytokine levels, it is possible that choline supplementation in children with FASD might also impact overall immune function [82,83]. For example, children and adolescents with FASD have increased rates of asthma and lung infections [84,85] and preclinical studies have shown that prenatal alcohol exposure disrupts immune function in the lungs [86,87,88]. Importantly, choline treatment reverses asthmatic conditions and reduces peripheral cytokines in an allergen-induced animal model [89]. Thus, choline can modify immune function in a number of ways. Given that altered immune function is observed in children with FASD, it will be critical to determine if choline also impacts the consequences of prenatal alcohol exposure for both immune function and health outcomes.

## 5. Conclusions

Data from the present study demonstrate that early choline treatment modifies cytokine levels, both at baseline and after an immune challenge, suggesting that early nutritional factors may have long-lasting effects on immune and neuroimmune functions. Importantly, choline also modified some of the effects of early alcohol exposure, even when administered after the alcohol insult. This suggests the possibility that the long-lasting cognitive improvements observed repeatedly in humans and animals perinatally supplemented with choline may be in part due to changing the inflammatory milieu of the hippocampus. Moreover, it will be important to determine if choline can successfully modify systemic immune responses and overall health outcome in individuals with FASD.

## Data Availability

The original contributions presented in the study are included in the article and Supplementary Materials. Further inquiries can be directed to the corresponding author (J.D.T.)

## Supplementary Materials

The following supporting information can be downloaded at: https://www.mdpi.com/article/10.3390/nu14142868/s1, Figure S1: Difference scores of hippocampal IL-13 levels in females and males following an immune challenge of LPS.
