# Supplementary figures and images for: Choline Supplementation Modifies the Effects of Developmental Alcohol Exposure on Immune Responses in Adult Rats
Source: Nutrients. 2022 Jul 13;14(14):2868. doi: 10.3390/nu14142868 (PMC9316525; doi:10.3390/nu14142868)

Hippocampus

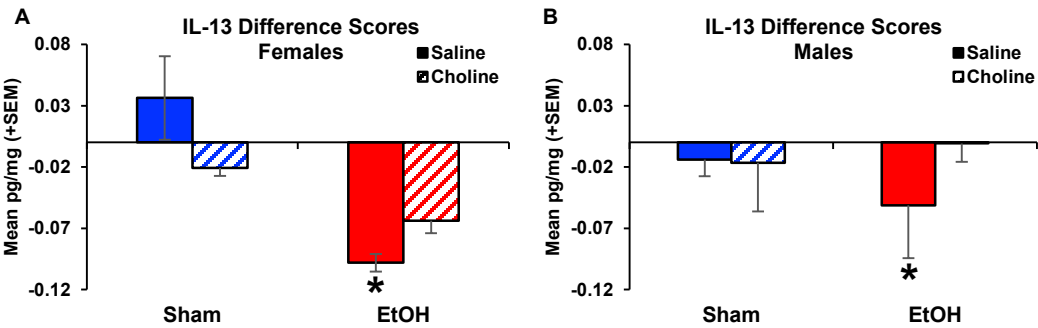

Figure S1

Supplement: Supplementary file 1 [file nutrients-14-02868-s001.zip › Supplementary Figure S1.pdf]
